# Supramolecular solvent-based microextraction for the preconcentration of Pb^2+^ and Cd^2+^ prior to spectrophotometric detection

**DOI:** 10.3906/kim-2106-24

**Published:** 2021-09-26

**Authors:** Huma ZAFAR, Faheem SHAH, Rafaqat Ali KHAN, Asad Muhammad KHAN, Jan NISAR, Bushra ISMAIL

**Affiliations:** 1 Department of Chemistry, COMSATS University Islamabad, Abbottabad Campus, Abbottabad, Pakistan; 2 National Center of Excellence in Physical Chemistry, University of Peshawar, Peshawar, Pakistan

**Keywords:** Supramolecular-solvent, lead, cadmium, microextraction, water

## Abstract

Supramolecular solvent-based dispersive liquid–liquid microextraction technique has been developed as a preconcentration tool for the determination of trace level of Pb^2+^ and Cd^2+^. Dodecanol dispersed in tetrahydrofuran has been utilized as a supramolecular-solvent system for the extraction of analytes prior to their quantitative determination with graphite furnace atomic absorption spectrophotometer. Both Pb^2+^ and Cd^2+^, which were efficiently extracted by supramolecular solvent system, were complexed with dithizone followed by the addition of supramolecular solvent. The experimental variables that could possibly influence the extraction efficiency, i.e. pH value, temperature, sample volume, centrifugation time, rate of centrifugation, ionic strength, etc. were subjected to the optimization step. An interference study was also conducted to check the selectivity of developed method. Limit of detection calculated for Pb^2+^ and Cd^2+^ was 0.015 and 0.061 mg L^−1^, respectively. The limit of quantification was 0.05 and 0.2 mg L^−1^ for Pb^2+^ and Cd^2+^, respectively. The analytical signal was enhanced to 30 times in case of Pb^2+^ and 27 times in case of Cd^2+^. The results obtained revealed that the developed method is rapid, simple, sensitive, and efficient for the determination of both analytes in real water samples.

## 1. Introduction

Lead (Pb^2+^) and Cadmium (Cd^2+^) are reported as potent pollutants for environment, and their toxic nature is a seriousconcern for ecological, environmental, nutritional, and evolutionary reasons [1]. These metals pose a great threat to human health because of high water solubility, non-biodegradability, and accumulation potential in different parts of the body[2]. These toxic heavy metals cause interference in normal body functions [3, 4]. Whereas Pb toxicity causes irreversibleeffects on health as there is no safe level or beneficial level of its exposure. Its toxicity predominantly affects renal, hepatic, reproductive, central nervous, and hematopoietic systems. It also badly affects bone and causes several disorders [5, 6].

Cd in trace amount is present in some foods like, seeds/grains, potatoes, leafy vegetables, mushrooms, crustaceans, liver, and kidney, etc. [7]. While major anthropogenic sources of Pb include occupational exposure, leaded gasoline, alloys, batteries (acid lead), plastics, solder, and cable sheeting, etc. [8, 9]. There are a number of analytical techniques available for heavy metal ions determination, such as inductively coupled atomic emission spectroscopy (ICP-AES), inductively coupled plasma optical emission spectroscopy (ICP-OES), X-ray fluorescence spectrometry (XRF), atomic fluorescence spectrometry (AFS) and atomic absorption spectrometry (AAS). However, AAS is considered most suitable for quantification of trace heavy metal ions as it is very efficient with good detection limits [10]. It is difficult to detect and quantify the trace-level concentration of toxic heavy metals in real samples with atomic absorption spectroscopic techniques, if their concentration is less than that of instrument’s detection limit. Moreover, interference also occur during the detection of the trace amounts from complex matrices. Hence, there is often a need for an appropriate pre-treatment step for the sample preparation. There are some drawbacks associated with the typical sample preparation methods such as a high chance of analyte loss because of multi-step procedures, time consuming and excessive use of (toxic) organic solvents [11].

Different techniques for the extraction and preconcentration of metals in different samples are available like, co-precipitation [12], solid phase extraction (SPE), liquid–liquid microextraction (LLME) etc., but these techniques utilizes a bulk volume of organic solvents [13]. One of the most efficient technique for metal preconcentration is dispersive liquid-liquid microextraction (DLLME) which is the modified form of liquid-liquid microextraction. Rezaee et al., in 2006, has proposed this technique for the first time. This recently developed technique relies on the formation of tiny droplets in sample solution as a consequence of rapid injection of solvent used for extraction (water immiscible) followed by vigorous dispersion of dispersive solvent (water miscible). This technique has been applied effectively for preconcentration of biological samples as well as environmental samples [14]. Rubio et al. reported a unique knotted reactor extraction technique for the simultaneous preconcentration of Cd and Pb in food samples. The developed technique was then compared with DLLME and found DLLME more efficient than knotted reactor extraction technique [15]. In another study, de Melo et al. reported vortex-assisted dispersive liquid-liquid microextraction for Pb and Cd determination. The developed method was successfully applied to sea water sample without any interference [16]. Werner developed deep eutectic solvent-based ultrasounds-assisted DLLME for same analytes. Application of the developed extraction technique showed excellent results in environmental sample [17].

Supramolecular solvent based dispersive liquid–liquid microextraction (SUPRAS-DLLME) technique is also derived from DLLME (based on the same principle) and has been utilized for preconcentration of Pb^2+^ and Cd^2+^. Supramolecular solvents are selected as extraction solvent to make the technique eco-friendlier. SUPRASs are the solvents with nano-structures produced from amphiphiles by a self-assembly process and shows immiscibility in water [18]. Non-covalent interactions exist among the molecules of solvent and have tremendous physicochemical properties rendering SUPRASs as attractive replacement of organic solvents used in analytical chemistry for extraction of different analytes (inorganic or organic). Extraction efficiencies mainly depend on binding energies that are established between SUPRASs and solutes (i.e. hydrogen bonding, dipole induced dipole, diploe-dipole and dispersion). As SUPRASs are made up of amphiphiles after an ordered arrangement in more assembled form, different regions become available with different polarities in SUPRASs. The hydrocarbon region (hydrophobic microenvironment) in SUPRASs is highly suitable for the extraction of organic or nonpolar compounds [19]. Nonflammability, nonvolatility, amphiphiles ubiquity in nature, tunable solvent properties, availability of regions of different polarities are among the few intrinsic properties of SUPRASs make them good substitutes of typically used toxic organic solvents [20, 21].

Nonionic surfactants are mainly utilized as SUPRASs for microextraction of heavy metals, which makes the technique cost effective, simple, rapid, and solvent consumption is greatly minimized. Therefore, this technique has been green alternatives to the classical liquid-liquid extraction techniques for the preconcentration of heavy metals have been developed and applied in this project.

## 2. Materials and methods

### 2.1. Apparatus

Spectrophotometric determinations were carried out with A-analyst 700 Perkin-Elmer atomic absorption spectrophotometer. For pH value measurement and adjustment, a pH meter (model HI-2210, purchased from Hanna instruments, UK) was used. Elmasonic sonicator (model E-30-H made by Elma-Hans Schmidbauer GmbH and Co) was utilized for sonication. Phase micro-syringe (5–500 microliters) model no; 1750 made by Hamilton. Co, USA was used for injection as well as for removal of extractant phase.

### 2.2. Chemicals and Reagents

All the chemicals and reagents used during the experimental work were of analytical grade. Lead and cadmium working standard solutions were prepared on daily basis by dilution of lead nitrate Pb(NO_3_)_2_ and cadmium nitrate Cd(NO_3_)_2_ standard stock solution (1000 ppm) (MERCK, Germany) in the double distilled water. Dithizone (C_13_H_12_N_4_S) was purchased from (Daejung, Chemical and Metal Co., Ltd. Korea) and used for chelation of lead and cadmium. Methanol (CH_3_OH) and Tetrahydrofuran (THF) obtained from (Daejung, Chemical and Metal Co., Ltd. Korea) where methanol was used to prepare dithizone stock solution and THF was used as disperser solvent. Dodecanol (Alfa Aesar, United States) was used as extraction solvent.

### 2.3. Extraction of Pb^2+^ and Cd^2+^ SUPRAS-DLLME procedure

First of all, 3 mL (20 ppm) standard solution of metal prepared in distilled water was taken in a centrifugation tube of 15 mL followed by the addition of 6 mL of ligand (0.01 M dithizone) solution. Then, 3 mL of sodium tetraborate buffer solution was added in order to make a stable metal ligand complex, and temperature was adjusted at 30–35 °C to get better recoveries. When metal ions reacted with the dithizone, hydrophobic complexes were formed that were easily extracted into the fine dispersed droplets. After that, optimal volume (0.90 mL) of extraction solvent, i.e. SUPRAS (1-dodecanol) was added along (0.70–0.80 mL) THF as dispersion solvent. The rapid injection of solvents was carried out by using 500 μL micro-syringe. These two solvents (dodecanol and THF) resulted in the formation of reverse micelles in aqueous medium, hence making the extraction of hydrophobic complexes easier. The solution was shaken by vortex mixer for 50 s to increase the interactions between SUPRAS system and the analytes. The solution became very turbid, and THF dispersed into the fine droplets in order to extract analyte. The resultant mixture was centrifuged for 8 min at 5000 rpm to carry out the phase separation. The viscous extractant layer was extracted through a micro-syringe, diluted, and analyzed through atomic absorption spectrometry.

## 3. Results and discussions

### 3.1. Selection of Extraction Solvent

**Figure 1:**
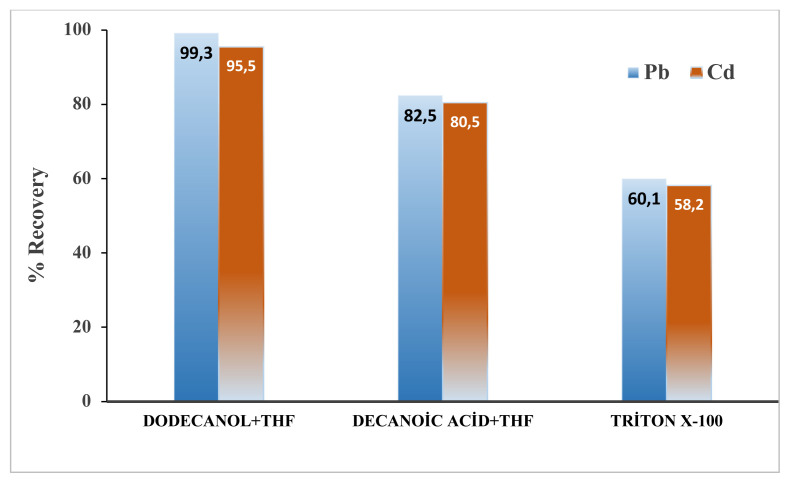
Effect of different SUPRASs on %Recovery of Pb^2+^ and Cd^2+^.

Extraction solvent has great significance in DLLME techniques as analyte’s extraction efficiency is greately infulenced by this crucial parameter [14]. Extraction solvent selection is mainly carried out by considering its physio-chemical properties, among which hydrophobicity is a characteristic, which is present in an ideal extraction solvent. Having great solubility for analyte compared to that in water is another important feature of ideal extraction solvent [22]. For selection of suitable solvent system, series of samples were prepared with different extraction solvents, and their effect on % recovery was observed. It is revealed, by Figure 1, that the minimum recoveries (60 % for Pb^2+^, 58 % for Cd^2+^) were obtained when extraction solvent was Triton X-100. When SUPRAS system of decanoic acid/THF was used as extraction solvent, the recoveries were less (82.5 % for Pb^2+^, 80.5 % for Cd^2+^) compared to those (99.3 and 95.5 % in case of Pb^2+^ and Cd^2+^, respectively) obtained when SUPRAS system of 1-dodecanol/THF was used. Therefore, SUPRAS system of 1-dodecanol/THF was used as extraction solvent.

### 3.2. Optimization of SUPRAS based DLLME Technique for preconcentration of Pb^2+^ and Cd^2+^

In order to improve the efficacy of method, optimization of several analytical parameters is required. Optimization of these parameters leads to high preconcentration factor and increased extraction efficiency even when the analyte concentration is very low. Therefore, various parameters were investigated, i.e. pH value, temperature, volume of extraction solvent, volume of dispersion solvent, centrifugation rate, centrifugation time, and sample volume to develop efficient SUPRAS-DLLME for Pb^2+^ and Cd^2+^.

#### 3.2.1. Effect of pH value

**Figure 2:**
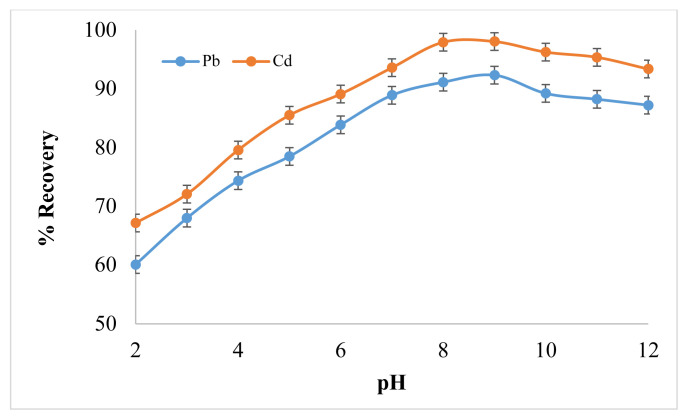
Effect of pH on Pb^2+^ and Cd^2+^ Analytical Response (N = 7).

pH value is a crucial factor that can greatly affect the extraction efficiency. Metal ion separation by microextraction technique involves prior complex formation, which would have enough hydrophobicity, so that it can be extracted in extractant phase [23]. It is more feasible to extract the analyte in its neutral form rather than its ionic form. Metal-ligand interactions for complex formation and extraction efficiency are pH dependent. Therefore, the pH effect on complex formation Pb(DHz)_2_ and Cd(DHz)_2_ was studied in the range of 2–12 by addition of different buffers. The maximum recovery of Pb^2+^ was obtained under slightly basic conditions at pH = 8, while the optimum value for Cd^2+^ recovery was attained at pH = 9. As shown in Figure 2, the extraction efficiency of Pb(DHz)_2_ in 1-dodecanol became maximized at pH = 8 and almost constant in the pH range of 10–12. Decrease in recoveries observed at lower pH value was probably because of competition between Pb^2+^ ion and H^+^ ion in reaction for the formation of complex with ligand [24]. The most probable reason behind less recovery of Cd^2+^ in acidic medium is that, in acidic medium, protonation is favored, while, in basic medium, deprotonation of ligand promotes the metal ligand complexation. Cd(DHz)_2_ can be easily extracted from slightly basic medium (pH = 9). In alkaline solution, when Cd^2+^ react with dithizone it forms neutral complex. The further study was carried out by keeping the pH value of sample at 8 for Pb^2+^ and pH 9 for Cd^2+^.

#### 3.2.2. Effect of Temperature and Incubation Time

**Figure 3:**
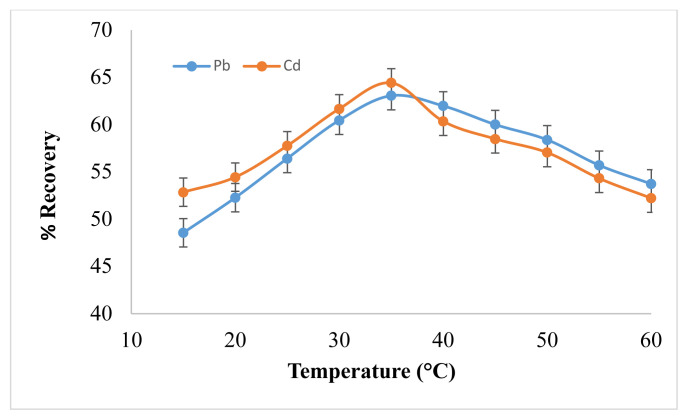
Effect of Temperature on Pb^2+^ and Cd^2+^ Analytical Response (N = 7).

An investigative study was carried out in which temperature range was selected from 15–60 °C with variable incubation time 5–20 min. The temperature affects the recovery directly from 15–30 °C and became maximum at 35 °C as depicted by Figure 3. While 10 min was found as most suitable incubation time, with further increase in temperature above 35 °C, the recovery started to decrease, resulting in less feasibility of both analytes trapped in the extracted layer due to which the analyte leached back in aqueous layer. Hence, analyte loss was observed in the form of decreased recovery percentage. Complex degradability at such high temperature can be another important reason for this decrease in % recoveries [25]. Therefore, 35 °C was selected as optimum temperature and 10 min as incubation time for further experimental work.

#### 3.2.3. Effect of Extractant and Disperser Volume

**Figure 4:**
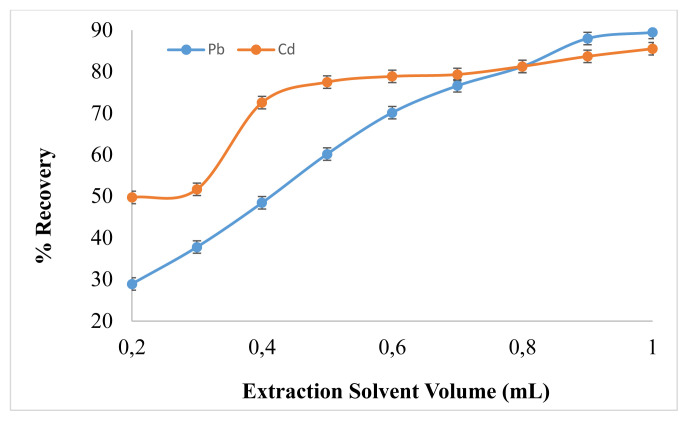
Effect of Extraction Solvent on Pb^2+^ and Cd^2+^ Analytical Response (N = 7).

Volume of extraction solvent (1-dodecanol) greatly affects the recovery of analyte. In order to obtain the ideal extractant volume, a study was carried out where different samples were prepared in which extraction solvent volume varied in a range of 0.2–1 mL. By increasing the extraction solvent volume, the recovery increases and became almost constant at 0.9–1 mL as shown in Figure 4. This means that below this value the volume of extraction solvent was insufficient for complete interaction of analyte and extractant molecules, hence, in the presence of sufficient extractant volume, high analytical response was attained for Pb^2+^ and Cd^2+^. The extraction of analyte was achieved by using SUPRAS (1-dodecanol-THF reverse micelles) because of hydrogen bonding and hydrophobic-hydrophobic interactions [26]. So, 0.9 mL extraction solvent volume was selected as optimum volume in further optimizations.

In any DLLME extraction procedure, dispersion solvent acts like a bridge for extractant-analyte interaction by dispersing the extraction solvent in aqueous analyte solution. The resulting turbid solution provides larger contact area between target analyte and the extractant, hence extraction efficiency get improved [27]. Miscibility of dispersion solvent is required in aqueous sample and extraction solvent. THF was selected as disperser solvent, and its effect on % recovery was studied in the range of 0.2–1.0 mL. The maximum recovery was obtained at 0.8 mL and 0.7 mL for Pb^2+^ and Cd^2+^, respectively. Below the optimum values, the recovery was lower as complete dispersion was not achieved. Thus, 0.80 and 0.70 mL was selected as an optimized dispersive solvent volume for extraction of Pb^2+^ and Cd^2+^.

#### 3.2.4. Effect of Sample Volume

**Figure 5:**
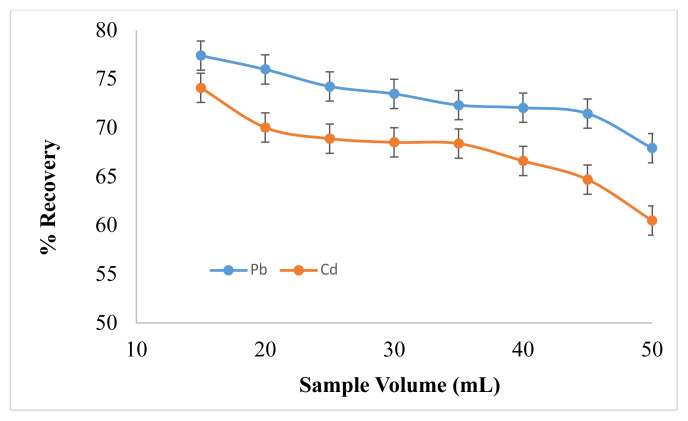
Effect of Sample Volume on Pb^2+^ and Cd^2+^ Analytical Response (N = 7).

Sample volume has a significant effect on the interactions among the target analyte and extractant. Its optimization is very important as sufficient interactions can be attained at an optimum sample volume. Sample volume was varied from 15–50 mL. As shown in Figure 5, when sample volume was less, the extraction recovery was maximum, but, when sample volume increased, the recovery started to decrease. The maximum quantitative recoveries obtained at 15 mL because, at this sample volume there is maximum interaction between analyte and extraction solvent. Whereas the most obvious reason behind decrease in recovery of analyte by increasing sample volume is that the analyte transfer would not be more pronounced from aqueous phase to extractant phase, resulting in less interactions between analyte and extraction solvent. Therefore, 15 mL was selected as optimized sample volume for further study.

#### 3.2.5. Effect of Centrifugation Time and Rate

In order to find out the time required to get maximum recovery by centrifugation, the study was carried out in the time range of (3–10 min). The recovery was maximum at 8 minutes and started to decrease with further increase in centrifugation time. The centrifugation rate was optimized in the range of 3000–10000 rpm and obtained 6000 rpm as optimum rate for Pb^2+^ and 5000 rpm for Cd^2+^. The decrease in recovery observed at higher centrifugation rate is due to the decomposition of extractant layer in which analyte is present. Hence, a centrifugation rate of 6000 rpm and time interval of 8 minutes was selected as optimized time for further study.

#### 3.2.6. Effect of NaCl

To study ionic strength influence on SUPRAS-DLLME performance for the preconcentration of Pb^2+^ and Cd^2+^, series of samples were prepared with varying concentrations of NaCl. 0.2–1.0 M solutions of NaCl were added in each sample while keeping the rest of analytical parameters constant. It was observed that by increasing the NaCl concentration there was not any significant increase in the recovery of analyte [25]. As in this project non-ionic surfactant has been used to develop SUPRAS system, so it did not get affected in term of efficiency by addition of NaCl [28]. Therefore, NaCl was not added in any sample for further studies.

### 3.3. Interference of coexisting ions

The efficiency of developed SUPRAS-DLLME method for preconcentration as well as for determination of Pb^2+^ and Cd^2+^ was studied in presence of several coexisting ions. Interfering ion is that ion which causes variation in analyte’s absorbance >5 % [26]. The results in Table 1 revealed that there is no pronounced effect of these coexisting ion on percentage recovery of analytes, hence these analytes can be determined successfully in complex matrices. Therefore, the efficacy of proposed method has been ascertained.

### 3.4. Validation of SUPRAS-DLLME through standard addition method

Prior to application, the developed SUPRAS-DLLME technique was validated through standard addition method. In this approach, known concentration of Pb^2+^ and Cd^2+^ standard solutions were added in tap water samples collected from the vicinity of COMSATS University Islamabad, Abbottabad Campus. The analytes under study were extracted and preconcentrated through SUPRAS-DLLME prior to atomic absorption spectrophotometric determination. Complex matrix effects were greatly reduced through SUPRAS-DLLME those cause interference with the analyte’s signal, hence maximum recoveries were obtained (Table 2). The recoveries obtained revealed the validity of developed technique. Furthermore, student’s t-test was applied, and it is found that there is no significant difference between added and found analytes amount at 95% confidence interval.

### 3.5. Analytical Figures of Merit

Analytical features of the developed method were calculated that characterized the performance of the developed method. These figures have great contribution towards method’s selectivity for analyte by manipulation of all the numerical parameters, which have characteristic importance. By keeping all the optimized parameters constant, the analytical figures of merit for SUPRAS-DLLME were calculated. Limit of detection and quantification, linear range, correlation coefficient, extraction recovery as well as enrichment and enhancement factor are important analytical figures. From the calibration curve, the linear equation and correlation coefficient for Pb^2+^ and Cd^2+^ are given in Table 3.

Another important figure of merit for a newly developed extraction procedure is the consumptive index (*CIn*). The efficacy of a preconcentration technique can also be assessed through its sample volume requirement. A good preconcentration technique should yield reliable and acceptable results even with limited sample volume [29].

The *CIn* can be defined as:


CIn=VsEF

where *Vs* is the sample volume utilized and *EF* is the enhancement factor [29]. Lower the value of *CIn*, efficient will be the preconcentration technique in terms of limited sample volume utilization. Numerical values of all these figures of merit were calculated and are summarized in Table 3.

### 3.6. Comparison with other reported techniques

Some of the analytical figures of merit of the proposed method have been compared to the literature reported microextraction/preconcentration techniques for the determination of both analytes (Table 4). It is evident that the analytical characteristics of the proposed technique are comparable with the reported techniques such as LOD, EF/PF, prompt and simple analysis compared to the advanced and expensive instrumentation. Furthermore, no tedious sample preparation steps were involved in the present proposed method as well as hazardous organic solvents have been utilized. The proposed preconcentration technique was found to a best alternative to the compared complex sample preparation techniques.

## 4. Conclusion

In this study, the proficiency of SUPRAS-DLLME technique for the extraction and preconcentration of Pb^2+^ and Cd^2+^ has been examined. The developed SUPRAS-DLLME technique was found to be the most suitable choice because it is cost effective, can withstand rough handling, and uses environmentally friendly chemicals. SUPRAS based extractants were found to be greener alternatives to the traditional organic solvents, as well as cost effective that ionic liquids. Low extractant volume resulted in high preconcentration factor with good recoveries. Furthermore, the proposed method is the best extraction tool for trace analytes in real samples with complex matrices and interferents.

## Figures and Tables

**Table 1:** Effect of interferants on Pb^2+^ and Cd^2+^ analytical response (N = 7).

Interferants	Added as	Tolerance level (mg L^−1^)	Recovery (%)	
			Pb^2+^	Cd^2+^
Li^+^	LiNO_3_	1000	96.1	97.2
Na^+^	Na_2_HPO_4_	2000	96.5	95.9
K^+^	K_2_SO_4_	2000	96.0	97.9
NH_4_^+^	NH_4_NO_3_	1000	98.3	97.1
Ca^2+^	CaSO_4_	1000	96.4	95.7
Cu^2+^	Cu(NO_3_)_2_	1000	97.1	97.4
Zn^2+^	Zn(CH_3_CO_2_)_2_	1000	96.9	97.2
Mg^2+^	MgCl_2_	1000	98.2	98.2
Al^3+^	Al(NO_3_)_3_	1000	96.7	96.8
Fe^3+^	Fe(NO_3_)_3_	1000	95.8	95.3
Cl^−^	MgCl_2_	2000	97.8	98.2
Br^−^	NaBr	1000	95.5	95.5
NO_3_^−^	Fe(NO_3_)_3_	3000	95.8	95.8
SO_4_^2−^	K_2_SO_4_	2000	95.9	98.1
CH_3_CO_2_^−^	Zn(CH_3_CO_2_)_2_	2000	97.1	96.8
HPO_4_^2−^	Na_2_HPO_4_	1000	96.5	95.4

**Table 2:** Validation of SUPRAS-DLLME for Preconcentration of Pb^2+^ and Cd^2+^.

Sample	Added amount (mg L^−1^)	Found amount (mg L^−1^)		% Recovery		t_Experimental_	
		Pb^2+^	Cd^2+^	Pb^2+^	Cd^2+^	Pb^2+^	Cd^2+^
Tap Water	0	BDL	BDL	-	-		
	10	10.02 ± 0.98^a^ (9.72 %)^b^	9.91 ± 1.11 (11.20 %)	100.2	99.1	0.097	0.812
	15	14.87 ± 2.45 (16.54 %)	14.78 ± 1.97 (13.32 %)	99.1	99.5	0.184	0.305
	20	18.02 ± 3.97 (22.03 %)	17.66 ± 4.02 (22.76 %)	90.1	98.3	1.782	1.594

BDL: Below detection limit.

a Standard deviation.

b Relative standard deviation.

t_critical_: 2.447, at 95 % confidence interval (p = 0.05)

**Table 3:** Analytical figures of merit calculated for SUPRAS-DLLME of Pb^2+^ and Cd^2+^.

Parameter	Pb^2+^	Cd^2+^
LOD (mg L^−1^)	0.015	0.061
LOQ (mg L^−1^)	0.05	0.2
ER (%)	97.02	96.73
PF	18.75	18.75
Linear Range (mg L^−^1)	0.05–20	0.2–20
Linear Equation (y= mx)	Y=0.18x	Y=0.689x
Correlation Coefficient (R^2^)	0.9996	0.9997
Enhancement Factor	30	27
CIn	0.51	0.55

LOD = Limit of Detection

LOQ = Limit of Quantification

% ER = % Extraction Recovery

PF= Preconcentration Factor

CIn = Consumptive Index

**Table 4:** Comparison of analytical characteristics of reported techniques to the proposed SUPRAS-DLLME of Pb^2+^ and Cd^2+^.

Sample	Preconcentration technique	Instrument	PF/EF		LOD		Sample volume (mL)	Reference
			Pb	Cd	Pb	Cd		
Cosmetics	SDIL-NDME	GFAAS	94.6	94.6	0.126 μgL^−1^	0.086 μgL^−1^	10	[30]
Plant sample	Hybrid imprinted polymer	FI-TS-FF-AAS	-	14	-	0.03 μgL^−1^	10	[31]
	Cloud point extraction	TS-FF-AAS	-		-	0.08 μgL^−1^	10	[32]
	Polyurethane foam minicolumn	FI-TS-FF-AAS	-	5	-	0.12 μgL^−1^	2	[33]
	Fullerene minicolumn	FI-TS-FF-AAS	-	11	-	0.10 μgL^−1^	1.5	[34]
Oil samples	DES-ME	FAAS	-	100	2.4 μgL^−1^	-	-	[35]
Honey	DLLME	FAAS	-	-	140 ngg^−1^	20 ngg^−1^	10	[36]
Human hair	Cloud point extraction	FAAS	43	22	2.86 μgL^−1^	0.62 μgL^−1^	10	[37]
Food samples	Solid phase extraction	FAAS	75	100	16.0 μgL^−1^	4.2 μgL^−1^	5	[38]
Sea water	Solid phase extraction	FAAS	-	-	28 μgL^−1^	6 μgL^−1^	10	[39]
Tap water	SUPRAS-DLLME	FAAS	97.02	96.73	0.015 mgL^−1^	0.061 mgL^−1^	15	present

SDIL-NDME: Single drop ionic liquid based non-dispersive microextraction.

GFAAS: Graphite furnace atomic absorption spectroscopy.

TS-FF-AAS: Thermospray flame furnace atomic absorption spectrometry.

DES-ME: Deep eutectic solvent microextraction.

DLLME: Dispersive liquid–liquid microextraction.

LC-UV: Liquid chromatography with UV detection.
